# Advancing Pediatric Cochlear Implant Care Through a Multidisciplinary Telehealth Model: Insights from Implementation and Family Perspectives

**DOI:** 10.3390/children13010039

**Published:** 2025-12-26

**Authors:** Chrisanda Marie Sanchez, Jennifer Coto, Jordan Ian McNair, Domitille Lochet, Alexandria Susan Mestres, Christina Sarangoulis, Meredith A. Holcomb, Ivette Cejas

**Affiliations:** 1 Department of Otolaryngology, University of Miami, Miami, FL 33136, USAmeredith.holcomb@med.miami.edu (M.A.H.);; 2 Department of Pediatrics, University of Miami, Miami, FL 33136, USA

**Keywords:** cochlear implants, pediatric audiology, telehealth, multidisciplinary care, remote programming

## Abstract

Background/Objectives: Multidisciplinary care is the gold-standard approach for delivering comprehensive pediatric healthcare. For children undergoing cochlear implant (CI) evaluation, multiple appointments are required to assess candidacy, set realistic expectations, and counsel families on rehabilitation and the psychosocial impact of hearing loss. Established pediatric CI users also need coordinated follow-up to address ongoing auditory, educational, and psychosocial needs. This study evaluated the satisfaction and family perspectives of the implementation of a virtual, team-based multidisciplinary model for both CI candidates and established CI users. Methods: Thirty-nine children and their families participated in discipline-specific telehealth consultations, including audiology, listening and spoken language (LSL) therapy, psychology, and educational services, followed by a 60 min multidisciplinary team meeting. Team meetings occurred during pre-implantation and at six months post-activation for CI candidates. Team meetings for established CI users were scheduled following completion of individual consultations. Providers summarized findings from their individual visits before transitioning to a caregiver-led discussion. Post-visit surveys assessed satisfaction and perceived benefit from the multidisciplinary model. Results: Thirty-nine dyads were enrolled (11 Pre-CI; 28 Established CI). Caregivers were predominantly mothers (89.7%), most identified as Hispanic (55.3%) and White (71.1%). Over half of children identified as Hispanic (59%) and White (71.8%); most were diagnosed with hearing loss at birth (55.9%). Satisfaction with the virtual model was uniformly high: 100% of caregivers were satisfied or very satisfied, and most rated care quality as “very good” or “excellent.” LSL therapy was most frequently rated as the most beneficial visit (70% Pre-CI; 45% Established CI). Caregivers strongly preferred ongoing team-based care, with 55–80% reporting that they would like it to occur every six months and 95–100% preferring remote meetings. Conclusions: A virtual multidisciplinary model offers a high-quality, family-centered approach for both CI evaluations and ongoing management of established CI users. By integrating simultaneous team-based sessions, this model not only supports the ‘whole child’ but also strengthens the family system by improving communication, streamlining care, and reducing the burden of multiple in-person appointments. Families consistently report high levels of satisfaction with the convenience, clarity, and collaboration provided through virtual team visits. Incorporating routine check-ins with families is essential to ensure their needs are addressed, reinforce progress, and guide timely, targeted interventions that maximize each child’s developmental outcomes.

## 1. Introduction

Children with hearing loss are at increased risk for delays in speech and language development [1,2,3,4,5], psychosocial functioning [6,7], educational outcomes [8,9,10], and overall quality of life [9,11]. Timely and appropriate intervention is essential to mitigate these risks. The Joint Committee on Infant Hearing (JCIH) recommends the 1-3-6 benchmark where children should be screened by 1 month, diagnosed by 3 months, and enrolled in early intervention by 6 months [12]. Effective care requires multiple appointments from diagnosis through follow-up. Many children with hearing loss also have comorbid medical conditions [13,14], further increasing the number of necessary visits. Innovative care models are needed to improve outcomes while reducing family burden.

The American Academy of Pediatrics identifies team-based care as the gold standard for managing pediatric healthcare [15]. Children with complex or chronic medical conditions often require care that addresses not only their primary diagnosis, but also the broader impact on development, behavior, and family well-being [16,17,18,19]. Multidisciplinary care integrates expertise from multiple healthcare professionals to create individualized, comprehensive care plans. Such models have demonstrated success in other pediatric fields, including craniofacial anomalies [20], oncology tumor boards [21], and pediatric oncology [22]. Although the literature in pediatric audiology is more limited, studies report high caregiver satisfaction with traditional in-person multidisciplinary models [23,24].

This study evaluates a telehealth-based model for managing pediatric hearing loss in children pre- or post-cochlear implantation (CI). Traditional CI teams include audiology, speech therapy, and otolaryngology. In our model, the team also includes psychology, listening and spoken language (LSL) therapy, and an educational specialist to provide more comprehensive support.

Telehealth allows families to access healthcare from home via electronic devices (e.g., laptop, cellphone). Interest in telehealth has grown substantially in recent years [25,26], particularly during the COVID-19 pandemic. With the expected increase in hearing loss [27] prevalence, efficient and scalable care models are needed. Prior studies show that telehealth can reduce in-person visits while maintaining outcomes, particularly for remote CI programming and audiology services [26,28,29]. However, these studies primarily focus on audiology and do not include the full multidisciplinary team needed for pediatric hearing loss management.

Despite the growing use of telehealth for audiologic services and remote CI programming, few studies have examined its feasibility or implementation within a truly comprehensive multidisciplinary framework that incorporates audiology, speech-language pathology, psychology, and educational services. These additional disciplines are essential for understanding the broader developmental, behavioral, emotional, and academic needs of children with hearing loss, needs that cannot be fully captured through audiologic testing alone. Psychologists offer expertise in socioemotional functioning and family well-being, while educational specialists evaluate classroom performance, communication access, and early learning skills. Traditionally, accessing this range of expertise requires families to attend multiple in-person appointments across different clinics and service systems, which creates substantial logistical challenges and makes coordination difficult for providers. A virtual delivery model has the potential to reduce these barriers by bringing the full team together in a single, accessible encounter. This approach supports more holistic assessment, more efficient care coordination, and earlier identification of developmental or educational concerns that may influence CI outcomes.

Accordingly, this study aimed to (1) evaluate the implementation of a fully remote, multidisciplinary CI team model for pediatric candidacy evaluations and for established CI recipients, and (2) examine caregiver satisfaction and perceived accessibility of telehealth-delivered multidisciplinary care.

## 2. Materials and Methods

### 2.1. Participants

Participants were assigned to one of two groups: children undergoing CI evaluation (Pre-CI group) and established CI recipients (Established CI group). For both groups, children were included if they were ages 6 months to 12 years old, had caregivers who agreed to the virtual visits, had access to a smartphone or computer, and who speak English and/or Spanish. For the Pre-CI group, children were enrolled once CI candidacy was confirmed using evidence-based practice guidelines on CI candidacy criteria. Children in the Established CI group were enrolled once they had at least 6 months of CI use. All families in the clinic who met the inclusion criteria were approached to participate in this study until the target enrollment number was met. Of note, the clinic serves a large tri-county area, including primarily individuals from diverse and minority backgrounds within both urban and suburban parts of the South Florida areas.

Forty-five participants (15 Pre-CI group, 30 Established CI group) were originally enrolled in the study. Four participants were excluded from data analysis in the Pre-CI group as they either did not proceed with CI or did not complete any study related measures. Two participants in the Established CI group were also excluded as they also did not attend the study visits or complete any measures. Thus, the sample being reported includes 39 pediatric CI candidates and established recipients (see Table 1 for participant demographics). This study was approved by the University of Miami IRB (IRB #20210584). Informed consent was obtained from all participants involved in the study.

### 2.2. Procedures

Participants were identified and approached by their clinical audiologist to participate in the multidisciplinary telehealth study. All participants completed discipline-specific telehealth consultations, which included audiology, LSL therapy, psychology, and educational services. Each consultation was conducted via a secure telehealth platform (Zoom) and focused on discipline-specific assessment and recommendations (see detailed explanations of each consultation below). Following the individual consultations, a virtual team meeting was scheduled. For established CI recipients, a multidisciplinary team meeting followed the completion of these individual sessions. For the Pre-CI group, team meetings were slated to occur pre-implantation and at 6 months post-activation. This study only reports the initial team meeting, as only 50% of the Pre-CI group attended the 6-month team meeting. All multidisciplinary team meetings were attended by the same core group of specialists, ensuring consistent representation from audiology, speech-language pathology, psychology, and education across all visits.

### 2.3. Audiology

All patients were initially seen in clinic for audiologic enrollment and baseline assessment. For the Pre-CI group, comprehensive evaluations were conducted, including audiometric testing and candidacy determination consistent with standard clinical protocols.

For the Established CI group, children attended their in-person routine visit and then were enrolled in the remote study. Follow-up audiology appointments were completed remotely and included device impedance measurements, electrically evoked compound action potentials (ECAPs), and assessment of overall audibility. These remote sessions additionally incorporated guided discussions between the audiologist, child, and caregiver to evaluate listening performance, device function, and progress toward auditory goals.

### 2.4. Listening and Spoken Language Therapy

Families in both the Pre-CI and Established CI groups participated in a remote check-in to discuss their child’s speech and language development and current concerns. When further evaluation was indicated, children were scheduled for a comprehensive speech and language assessment either in person or via telehealth using a standard protocol. Assessment sessions followed the same structure and duration as in-person visits—60 min for younger children under 2 years and 120 min for those aged 3 and older. Younger children’s evaluations included parent interviews, listening activities, and language observation, while older children’s sessions included age-appropriate speech and language assessments. All sessions were conducted by a certified listening and spoken language specialist (LSLS Cert. AVT) using evidence-based auditory-verbal strategies in the child’s primary language (English, Spanish, or both). For bilingual assessments, therapists followed tele-practice guidelines from each test publisher to ensure standardized administration. Both English and Spanish assessments were administered by speech-language pathologists with documented Spanish fluency and competency, following test manuals to ensure fidelity and accuracy across languages.

### 2.5. Education Consultation

Each family participated in a consultation via phone or a virtual platform with a deaf and hard-of-hearing (D/HH) educational specialist as part of the multidisciplinary CI evaluation process. Families were asked to provide relevant educational documentation (e.g., Individualized Education Programs [IEPs], Individualized Family Service Plans [IFSPs], or 504 plans) prior to the meeting. During the consultation, the D/HH specialist reviewed the child’s educational history, current placement, and academic progress, with discussions tailored according to whether the child was in the Pre-CI Group or Established CI Group. The D/HH specialist identified areas of educational need, provided counseling regarding school-based support, and reviewed appropriate accommodations and interventions aligned with the child’s communication and listening goals.

When children lacked existing educational support, discussions focused on available resources and strategies to initiate services, as well as appropriate educational placements. For those with established plans, the discussion focused on optimizing current support and addressing ongoing challenges. Each session concluded with a summary of recommendations and key points to be communicated to the multidisciplinary CI team.

### 2.6. Psychology Consultation

Psychology consultations were conducted remotely and lasted approximately 30 to 60 min. The primary purpose of these visits was to assess each child’s functioning across academic, social, emotional, and behavioral domains. When no current concerns were reported by the family, the consultation served as an opportunity to provide education on the available psychology services and how to access them in the future if needed. When parents or caregivers identified concerns related to their child’s behavior, mood, learning, or adjustment, additional information was obtained to clarify the nature and extent of the difficulties. Based on this initial screening, follow-up services were arranged as appropriate, including individual or family therapy, or a comprehensive psychoeducational evaluation to assess potential learning, attention, or neurodevelopmental disorders.

### 2.7. Multidisciplinary Team Meeting

Multidisciplinary team meetings were held via Zoom for approximately 60 min and included the audiologist, LSLS Cert. AVT speech-language pathologist, psychologist, educational specialist, and parent. During the team visit, findings from each provider were reviewed in the context of the child’s broader medical, audiological, developmental, and therapeutic care. Providers briefly summarized findings and recommendations from their individual consultations, after which the discussion shifted to a parent-led discussion to address priorities and concerns. Based on the multidisciplinary discussion, individualized recommendations were formulated, and appropriate referrals for follow-up services were provided as indicated. At the conclusion of the visit, parents were invited to complete a brief survey evaluating their satisfaction with the multidisciplinary telehealth experience, including perceived coordination of care, communication among providers, and the relevance of recommendations to their child’s needs (all survey questions and responses can be found in the Supplemental Table S3a–c).

### 2.8. Virtual Team Visit Satisfaction

Caregivers in both cohorts completed post-visit surveys assessing the acceptability of and satisfaction with the virtual multidisciplinary model. A brief family satisfaction survey was administered through Qualtrics to evaluate caregiver satisfaction of the virtual multidisciplinary team visit. The eight-item survey included a mix of response formats, including multiple-choice questions and Likert-scale ratings (see Table 1). Families accessed and completed the survey directly through the Qualtrics platform, which could be completed on a computer, tablet, or smartphone at their convenience with a unique survey access code. This format allowed flexible participation and ensured that responses were collected in a secure, standardized manner. All questions from the Virtual Team Visit with their results can be found in Supplemental Table S3a–c.

## 3. Results

### 3.1. Demographics

A total of 39 parent–child dyads participated in the study (11 Pre-CI and 28 in Established CI). In the Pre-CI group, eleven patients (54.5% female) with a mean age of 4.29 years (SD = 4.15) and their caregivers (all mothers) were enrolled. Caregivers had a mean age of 33.11 years (SD = 5.28); 63.6% were married and most identified as Hispanic (54.5%) and/or White (72.7%). Nearly half (45.5%) reported holding a graduate degree, and 27.3% were working on-site at the time of the study. Among the children, 54.5% identified as Hispanic and 63.6% as White. Over half (54.5%) were diagnosed with hearing loss at birth, all had been fit with hearing aids, and 63.6% received their initial hearing aid fitting after 12 months of age.

In the Established CI group, twenty-eight patients (42.9% female) with a mean age of 6.43 years (SD = 3.16) and their caregivers (85.7% mothers) were enrolled. Caregivers had a mean age of 38 years (SD = 9.13), and the majority were married (59.3%). Most caregivers identified as Hispanic (55.6%) and/or White (70.4%). The most reported highest education level was a bachelor’s degree (33.3%), and 44.4% were working on-site.

Among the children, 63% identified as Hispanic and 74.1% as White. Most were diagnosed with hearing loss at birth (55.6%), and 88.9% had been fit with hearing aids. Hearing aid fitting typically occurred between 6 and 12 months of age (34.8%) or after 12 months (34.8%). Further demographic information for the Established CI group is presented in Table 2.

Full demographic information is presented in Table 2.

### 3.2. Survey Results

A subset of participants missed the team meeting; thus, the survey results reflect the number of those who attended. In the Pre-CI group, four additional enrolled participants (26.6% of the total) were excluded due to incomplete surveys or not proceeding with CI. In the Established CI group, two participants were excluded for missing most appointments, and five of the remaining 28 missed the team meeting (17.8%).

Given the small sample size and limited variability in responses, all survey data are presented using descriptive statistics. Comparative statistical testing (e.g., chi-square, t-test) was not appropriate, as several contingency cells contained very low counts, which could violate assumptions and produce unstable estimates. Descriptive measures, including frequencies, percentages, means, and standard deviations, provide an accurate summary of caregiver responses and observed trends across the Pre-CI and Established CI groups. All questions and results can be found in Supplemental Tables S1–S3.

### 3.3. Technology Use

In the Pre-CI group, most caregivers reported using the following on a daily basis: a smartphone (100%) or computer/tablet (90%); checking email (100%), sending emails (70%), and sending text messages (90%). Half of caregivers reported regularly using a video conferencing service (50%) or using Bluetooth technology to pair their devices (80%). Four caregivers disagreed that people close to them would refer to them as “tech savvy” (40%) and one caregiver agreed that they are easily frustrated by technology (10%).

Similarly, in the Established CI group, most caregivers reported daily smartphone use (91.3%) and more than half used a computer or tablet each day (65.2%). Regular communication tasks were also common, including checking email (91.3%), sending emails (65.2%), and texting (87%). Many caregivers routinely used video-conferencing platforms (69.6%) and paired devices via Bluetooth (78.2%). Despite this frequent technology use, 43.5% of caregivers disagreed that people close to them would describe them as “tech savvy,” and one caregiver (4.3%) strongly agreed that they are easily frustrated by technology.

Overall, both groups demonstrated sufficient technology skills to participate effectively in virtual multidisciplinary visits. All participants scheduled for the virtual team meeting were able to access the session without technical issues. All technology questions and results are highlighted in Supplemental Table S1.

### 3.4. Visit Time Burden

Caregivers in both groups generally reported that attending appointments was manageable, though ease differed between groups. In the Pre-CI group, most caregivers agreed or strongly agreed that it was easy to attend their appointment (81.8%) and to arrive on time (63.6%). In contrast, just over half of caregivers in the Established CI group agreed or strongly agreed that attending their appointment (51.9%) and arriving on time (51.9%) was easy.

Time off from work also varied by group. In the Pre-CI group, half of caregivers reported taking some time off work, with 54.5% reporting no time off, 9.1% taking 2–4 h, 18.2% taking 4–6 h, and 18.2% taking 6–8 h; 60% of caregivers used unpaid time off. Among the Established CI group, most caregivers did not take time off (85.7%), while 10.7% took 4–6 h and 3.6% took 6–8 h; 75% of caregivers used paid time off.

Regarding patients, three in the Pre-CI group (27.3%) and five in the Established CI group (17.9%) had to take time off school to attend the appointment. Additionally, half of Pre-CI caregivers (50%) and 14.3% of Established CI caregivers had to coordinate with someone else to watch other children or dependents. All Visit Time Burden questions and their results are highlighted in Supplemental Table S2a.

### 3.5. Caregiver Satisfaction

Caregiver satisfaction with the clinic visit was high in both groups. All caregivers in both the Pre-CI and Established CI groups reported being “satisfied” or “very satisfied” with their visit. Most caregivers in the Pre-CI group rated the quality of care as “very good” or “excellent” (90.9%), while all caregivers in the Established CI group rated the quality of care as “very good” or “excellent” (100%). Similarly, the majority of caregivers reported that their provider spent “just the right” amount of time with them: 90.9% in the Pre-CI group and 96.3% in the Established CI group. All Caregiver Satisfaction questions and their results are highlighted in Supplemental Table S2b.

### 3.6. Multidisciplinary Team Meeting

**Figure 1:**
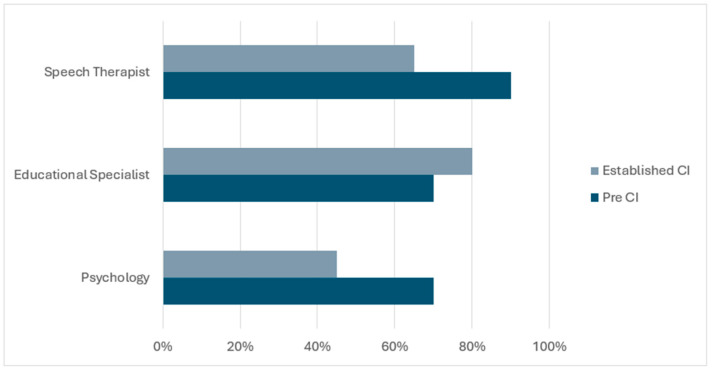
Which specialists would you like to see again?

For multidisciplinary team meetings, caregivers in both groups endorsed high satisfaction and perceived importance of collaboration. In the Pre-CI group, all caregivers were “extremely satisfied” (100%) and endorsed that it was “extremely” important for all their child’s providers to work together. Speech therapy was identified as the most beneficial visit (70%), followed by psychology (20%) and educational specialist (10%). Most caregivers wanted to see all specialists again, including the speech therapist (90%), psychologist (70%), and educational specialist (70%; See Figure 1). Caregivers preferred follow-up every six months, representing 80% of the group, favored remote meetings, representing 100%, and preferred team-based meetings at 90% rather than individual specialist visits.

In the Established CI group, 95% of caregivers were “extremely satisfied” with the multidisciplinary team meeting and 5% were “somewhat satisfied.” All caregivers endorsed that provider collaboration was “extremely” important. Speech therapy was identified as the most beneficial (45%), followed by educational specialist (35%) and psychology (20%; See Figure 1). Caregivers wanted to see all specialists again, including the educational specialist (80%), speech therapist (65%), and psychologist (45%). Caregivers preferred follow-up every six months, representing 55% of the group, favored remote meetings at 95% and half preferred team-based meetings while the other half were equally willing to meet with individual specialists.

Comparative observations between the groups revealed that Pre-CI caregivers placed greater emphasis on LSL therapy as the most beneficial (70% vs. 45% in Established CI) and were more likely to prefer seeing all specialists again. In contrast, caregivers in the Established CI group placed relatively greater emphasis on the educational specialist visits (35% vs. 10%). Follow-up preferences also differed: Pre-CI caregivers more frequently preferred six-month intervals (80% vs. 55%) and team-based meetings (90% vs. 50%), whereas half of the Established CI cohort were equally willing to meet with individual specialists. Despite these differences, satisfaction ratings and the perceived importance of provider collaboration remained consistently high across both groups. All Multidisciplinary Team Visit satisfaction and preference questions and their results are highlighted in Supplemental Table S3a–c.

Overall, virtual multidisciplinary CI care was associated with high caregiver satisfaction, adequate technology use, and manageable logistical burden. Patterns differed across Pre-CI and Established CI groups, highlighting the importance of stage-specific, tailored multidisciplinary support.

Figure 1 highlights which specialists each group preferred seeing again following their virtual visit excluding audiology (as this is considered a required specialty for pediatric CI care).

## 4. Discussion

This study evaluated the implementation of a virtual multidisciplinary model for pediatric CI evaluation and follow-up care. We also assessed caregiver satisfaction and accessibility of telehealth services. Our findings show that this approach can effectively integrate multiple disciplines into a coordinated virtual format, maintaining quality of care while enhancing family engagement.

Nearly all caregivers reported being “very satisfied” with the team-based telehealth format and rated the quality of care as “excellent.” High satisfaction with virtual delivery and desire for regular team meetings highlight the value of this model in improving accessibility and reducing the logistical burden of care. These results support telehealth as a promising strategy to address access disparities and improve efficiency in pediatric CI care.

Traditional multidisciplinary CI care typically requires multiple in-person appointments over several days or weeks [30]. While effective, these models can create challenges for families, including travel time, missed work, and coordination with school schedules [31,32]. The virtual model consolidates multiple provider perspectives into a single meeting, reducing the in-person visits without sacrificing quality of care. This aligns with previous research showing that remote CI programming can achieve high patient satisfaction and outcomes comparable to in-person services [26].

Telehealth offers unique advantages for pediatric CI programs. It reduces travel burden, increases access for families living far from implant centers, and allows for the inclusion of caregivers or educational team members who may not regularly attend in-person appointments [26,29]. This model also promotes real-time interdisciplinary communication, ensuring consistency in recommendations and care plans [33]. Strong parental satisfaction for remote delivery suggests that virtual models may support long-term engagement with the CI team, potentially improving adherence to rehabilitation plans and follow-up schedules.

This study also highlights the value of proactive multidisciplinary care. Families can access specialized expertise before concerns become critical. Traditionally, specialties such as psychology and education are engaged only when problems arise. In this model, remote team meetings allowed early identification and discussion of developmental, behavioral, or educational needs. Families reported high satisfaction with this proactive approach, with most expressing interest in ongoing follow-up sessions, reflecting that they viewed coordinated multidisciplinary input as a useful component of their child’s ongoing care, even if no specific concerns were present at the time. Subgroup differences further emphasize the importance of individualized care. Families of children in the Established CI group often identified the educational component as the most beneficial and wanted continued follow-up. Contrarily, families in the Pre-CI group most frequently valued LSL services. These differences reflect developmental stages of children and clinical priorities: children with established CI focus on educational performance, while those undergoing CI evaluation are developing auditory and spoken language skills. These findings reinforce the need to tailor multidisciplinary discussions to each child’s developmental level and stage in the intervention process.

Families also rated the final team meeting positively. Survey items specifically asked caregivers how well the multidisciplinary team appeared “on the same page” and how important it was for their child’s providers to work together. Caregivers reported that hearing a unified message from multiple specialists clarified next steps, minimized confusion, and strengthened confidence in the care plan. This collaborative wrap-up appears to be a key feature of the model, enhancing family understanding, satisfaction, and perceived continuity of care. These findings align with research conducted by Albertson and colleagues [34] demonstrating that clear and coordinated messages from the care team enhance caregivers’ understanding and overall satisfaction.

## 5. Limitations

Despite these advantages, several challenges remain. While our findings suggest that virtual multidisciplinary CI care is feasible and well-received, the study’s single-center design and relatively small sample limit generalizability. Further research across multiple sites with larger samples is needed to confirm these results and support broader implementation. Telehealth delivery depends on reliable internet, access to devices, and caregiver familiarity with virtual platforms. While most participants reported regular technology use, the cohort may underrepresent families with limited access, lower digital literacy, or constrained socioeconomic resources, who may face additional barriers to participating fully in virtual care. Addressing these digital and socioeconomic factors is critical to ensuring equitable access, and future studies should explore strategies such as device or internet provision, caregiver training in telehealth platforms, and flexible scheduling to support diverse populations. Some assessments, particularly in speech and language, required modifications for remote administration, and not all tools are formally validated for telemedicine. Certain audiologic care tasks, such as device troubleshooting or programming adjustments, still require in-person visits.

Billing and reimbursement also present important considerations for broader implementation. Because this project was conducted as a research study, the virtual multidisciplinary visits were not billed to insurance, and no clinical billing codes were applied. In standard clinical settings, however, reimbursement remains a significant barrier. Existing payer structures often do not support integrated, multi-provider telehealth encounters. For example, commonly used telehealth evaluation and management codes, time-based behavioral health codes, and therapy service codes are typically structured for single-provider visits and may not allow simultaneous or sequential same-day billing across multiple disciplines. Some insurers also restrict reimbursement for extended multidisciplinary case discussions or require in-person components for coverage. These limitations may constrain scalability in routine care. Establishing feasible billing pathways and evaluating the cost-effectiveness of multidisciplinary telehealth models will be essential for sustainable implementation beyond the research context.

Finally, a subset of participants (15.6%) missed several appointments, including the final team meeting. Reasons for missed visits were unknown, highlighting ongoing challenges in coordinating multidisciplinary care. Despite these limitations, structured proactive check-ins provide meaningful benefits for families who seek to engage with the full team.

### Future Directions

Our results support broader adoption of virtual multidisciplinary care for pediatric CI programs. Future work should validate remote assessment protocols, expand access to remote programming technologies, and explore reimbursement models to support sustainability. Multi-center trials could evaluate scalability and outcomes across populations and health systems. Integrating virtual multidisciplinary care into standard practice has the potential to improve access, enhance family-centered care, and optimize long-term outcomes. This project underscores the value of a comprehensive multidisciplinary team, including audiology, LSL therapy, psychology, and education. Structured “check-in” visits allow teams to identify needs proactively. In many cases, children categorized as “good performers” did not report any notable concerns; yet challenges emerged and became apparent when directly assessed by the team or through surveys and discussion. Routine check-ins, whether virtual or in person, help uncover unmet needs, align recommendations, and prompt timely referrals.

Overall, virtual multidisciplinary CI care improves access, achieves high family satisfaction, and identifies needs that vary across the implant journey. Differences between evaluation and post-implant groups highlight the importance of adaptable, stage-specific care models. Addressing ongoing barriers such as reimbursement and telehealth validation will be essential for wider adoption. These findings support a scalable, sustainable, and family-centered framework for virtual multidisciplinary CI management.

## 6. Conclusions

A virtual multidisciplinary CI care model unites core pediatric hearing healthcare specialists in a single coordinated session, reducing travel and scheduling burdens while enhancing team communication. Families report high satisfaction with this collaborative, streamlined approach, which enables earlier identification of needs and clearer guidance throughout the CI journey. Together these strengths highlight virtual multidisciplinary care as a scalable, equitable, and family-centered strategy for delivering high-quality CI services.

## Figures and Tables

**Table 1:** Questions from the Remote Multidisciplinary Satisfaction Visit questionnaire.

1.	Which of your remote visits with the multi-disciplinary team was most beneficial for you? (Choose one)
2.	How satisfied were you with the overall multi-disciplinary team meeting?
3.	How important is it for your child’s providers to work together?
4.	How well do you think the entire team was on the same page about your child’s care?
5.	Which specialists would you like to see again? (Select all)
6.	How often would you like to meet with the multidisciplinary team?
7.	Would you prefer to meet with the multi-disciplinary team members individually or as a group?
8.	Would you want these visits to be remote or in-person?

**Table 2:** Sample Characteristics.

	Pre-CI *n* = 11	Established CI *n* = 28
**Characteristic**	**Mean (*SD*)**	**Mean (*SD*)**
Child Age	4.29 *(4.15)*	6.43 *(3.16)*
Caregiver age	33.11 *(5.28)*	38.00 (9.13)
**Characteristic**	***n*** **(%)**	***n (%)***
**Caregiver relationship to child**		
Mother	11 (100%)	24 (85.7%)
Father	0 (0%)	4 (14.3%)
**Caregiver marital status**		
Married	7 (63.6%)	16 (59.3%)
Separated	0 (0%)	3 (11.1%)
Living with partner	3 (27.3%)	1 (3.7%)
Single	1 (9.1%)	7 (25.9%)
**Caregiver race**		
White	8 (72.7%)	19 (70.4%)
Black	3 (27.3%)	5 (18.5%)
Asian	0 (0%)	1 (3.7%)
Other	0 (0%)	2 (7.4%)
**Caregiver ethnicity**		
Non-Hispanic	5 (45.5%)	12 (44.4%)
Hispanic	6 (54.5%)	15 (55.6%)
**Caregiver highest education**		
Less than high school	1 (9.1%)	0 (0%)
High school	2 (18.2%)	1 (3.7%)
Technical school	1 (9.1%)	1 (3.7%)
Some college/no degree	0 (0%)	4 (14.8%)
Associate’s degree	1 (9.1%)	6 (22.2%)
Bachelor’s degree	1 (9.1%)	9 (33.3%)
Graduate degree	5 (45.5%)	6 (22.2%)
**Caregiver employment status**		
Unemployed	1 (9.1%)	5 (18.5%)
Working from home	2 (18.2%)	4 (14.8%)
Self-employed	1 (9.1%)	0 (0%)
Working on site	3 (27.3%)	12 (44.4%)
Combination: home/on site	2 (18.2%)	3 (11.1%)
Homemaker	2 (18.2%)	3 (11.1%)
**Child Sex**		
Female	6 (54.5%)	12 (42.9%)
Male	5 (45.5%)	16 (57.1%)
**Child Race**		
White	7 (63.6%)	20 (74.1%)
Black	3 (27.3%)	4 (14.8%)
Asian	0 (0%)	1 (3.7%)
Other	1 (9.1%)	2 (7.4%)
**Child Ethnicity**		
Non-Hispanic	5 (45.5%)	10 (37.0%)
Hispanic	6 (54.5%)	17 (63.0%)
**Child diagnosed with hearing loss**		
At birth	6 (54.5%)	15 (55.6%)
Before 3 months	0 (0%)	4 (14.8%)
Between 6 and 12 months	0 (0%)	2 (7.4%)
Other age	5 (45.5%)	6 (22.2%)
**CI recommended (age)**		
Before 3 months	1 (9.1%)	1 (3.7%)
Between 3 and 6 months	0 (0%)	6 (22.2%)
Between 6 and 12 months	3 (27.3%)	5 (18.5%)
Other age	7 (63.6%)	15 (55.6%)
**Child fit with hearing aids**		
Yes	11 (100%)	24 (88.9%)
No	0 (0%)	3 (11.1%)
**Fit with hearing aids (age)**		
Before 3 months	0 (0%)	1 (4.3%)
Between 3 and 6 months	4 (36.4%)	6 (26.1%)
Between 6 and 12 months	0 (0%)	8 (34.8%)
Other age	7 (63.6%)	8 (34.8%)

## Data Availability

The original contributions presented in this study are included in the article/Supplementary Materials. Further inquiries can be directed to the corresponding author.

## Supplementary Materials

The following supporting information can be downloaded at: https://www.mdpi.com/article/10.3390/children13010039/s1. Table S1: Caregiver Technology Use and Comfort Questions and Results. Table S2a: Time, Work, School, and Care Coordination Burden Questions and Results. Table S2b: Caregiver Satisfaction with In-Person Clinic Visit Questions and Results. Table S3a: Multidisciplinary Team Satisfaction Questions and Results. Table S3b: Caregiver Preferences for Multidisciplinary Team Questions and Results. Tables S3c: Caregiver Preferences for Multidisciplinary Team Follow-Up Questions and Results.

## References

[1] Geers A.E., Nicholas J.G. (2013). Enduring advantages of early cochlear implantation for spoken language development. J. Speech Lang. Hear. Res..

[2] Dettman S.J., Dowell R.C., Choo D., Arnott W., Abrahams Y., Davis A., Dornan D., Leigh J., Constantinescu G., Cowan R. (2016). Long-term communication outcomes for children receiving cochlear implants younger than 12 months. Otol. Neurotol..

[3] Stika C.J., Eisenberg L.S., Carter A.S., Johnson K.C., Ganguly D.M.H., Henning S.C., DesJardin J.L. (2021). Developmental outcomes in Early-Identified children who are hard of hearing at 2 to 3 years of age. Ear Hear..

[4] Markman T.M., Quittner A.L., Eisenberg L.S., Tobey E.A., Thal D., Niparko J.K., Wang N. (2011). Language development after cochlear implantation: An epigenetic model. J. Neurodev. Disord..

[5] Ching T.Y.C., Dillon H. (2013). Major findings of the LOCHI study on children at 3 years of age and implications for audiological management. Int. J. Audiol..

[6] Wong C.L., Ching T.Y.C., Cupples L., Button L., Leigh G., Marnane V., Whitfield J., Gunnourie M., Martin L. (2017). Psychosocial development in 5-Year-Old children with hearing loss using hearing aids or cochlear implants. Trends Hear..

[7] Polat F. (2003). Factors affecting psychosocial adjustment of deaf students. J. Deaf Stud. Deaf Educ..

[8] Teasdale T.W., Sorensen M.H. (2007). Hearing loss in relation to educational attainment and cognitive abilities: A population study. Int. J. Audiol..

[9] Cejas I., Barker D.H., Petruzzello E., Sarangoulis C.M., Quittner A.L. (2023). Cochlear implantation and Educational and Quality-of-Life Outcomes in Adolescence. JAMA Otolaryngol.–Head Neck Surg..

[10] Idstad M., Engdahl B. (2019). Childhood Sensorineural hearing loss and Educational Attainment in Adulthood: Results from the HUNT study. Ear Hear..

[11] Haukedal C.L., Lyxell B., Wie O.B. (2019). Health-Related Quality of Life with Cochlear Implants: The Children’s Perspective. Ear Hear..

[12] American Academy of Pediatrics, Joint Committee of Infant Hearing (2020). Year 2019 position statement: Principles and guidelines for early hearing detection and intervention programs. J. Early Hear. Detect. Interv..

[13] Olivier N., Shepherd D.A., Smith L., Carew P., Paxton G.A., Downie L., Rose E., Dawes K., Sung V. (2022). Etiology, comorbidities, and health service use in a clinical cohort of children with hearing loss. Ear Hear..

[14] Wainwright E., Pham H., Clack R., Irvine N., Tran T.T., Maywood E., Rodrigues S., Hird K., Kuthubutheen J. (2025). Detailed prevalence analysis of medical co-morbidities in paediatric cochlear implant recipients. Int. J. Pediatr. Otorhinolaryngol..

[15] Katkin J.P., Kressly S.J., Edwards A.R., Perrin J.M., Kraft C.A., Richerson J.E., Tieder J.S., Wall L., Alexander J.J., Flanagan P.J. (2017). Guiding Principles for Team-Based Pediatric care. Pediatrics.

[16] Leach K.F., Stack N.J., Jones S. (2021). Optimizing the multidisciplinary team to enhance care coordination across the continuum for children with medical complexity. Curr. Probl. Pediatr. Adolesc. Health Care.

[17] Children and Youth with Special Health Care Needs (CYSHCN)|MCHB. https://mchb.hrsa.gov/programs-impact/focus-areas/children-youth-special-health-care-needs-cyshcn.

[18] Zonta J.B., Okido A.C.C., De Lima B.J., Martins B.A., Looman W.S., Lopes-Júnior L.C., Silva-Rodrigues F.M., De Lima R.a.G. (2024). Stress in Family Caregivers of Children with Chronic Health Conditions: A Case–Control Study. Children.

[19] Cohn L.N., Pechlivanoglou P., Lee Y., Mahant S., Orkin J., Marson A., Cohen E. (2020). Health Outcomes of Parents of Children with Chronic Illness: A Systematic Review and Meta-Analysis. J. Pediatr..

[20] Robin N.H., Baty H., Franklin J., Guyton F.C., Mann J., Woolley A.L., Waite P.D., Grant J. (2006). The multidisciplinary evaluation and management of cleft lip and palate. South. Med. J..

[21] Wheless S.A., McKinney K.A., Zanation A.M. (2010). A prospective study of the clinical impact of a multidisciplinary head and neck tumor board. Otolaryngol.-Neck Surg..

[22] Cantrell M.A., RubleRN C.K. (2011). Multidisciplinary care in pediatric oncology. J. Multidiscip. Healthc..

[23] Findlen U.M., Malhotra P.S., Adunka O.F. (2018). Parent Perspectives on Multidisciplinary Pediatric Hearing Healthcare. Int. J. Pediatr. Otorhinolaryngol..

[24] Hawley K.A., Goldberg D.M., Anne S. (2017). Utility of a Multidisciplinary Approach to Pediatric Hearing Loss. Am. J. Otolaryngol..

[25] Evans T., Nejman T., Stewart E., Windmill I. (2021). Increasing pediatric audiology services via Telehealth. Semin. Hear..

[26] Holcomb M.A., Coto J., Stern T., Sarangoulis C.M., Cejas I., Sanchez C.M. (2025). Remote care: The future of cochlear implants. Otol. Neurotol..

[27] World Health Organization: WHO Deafness and Hearing Loss. https://www.who.int/news-room/fact-sheets/detail/deafness-and-hearing-loss.

[28] Holcomb M.A., Smeal M.R. (2024). How to teach an “Old dog” new tricks: Improving Clinical Efficiency in a Well-Established Cochlear Implant Program. Otol. Neurotol..

[29] Slager H.K., Jensen J., Kozlowski K., Teagle H., Park L.R., Biever A., Mears M. (2019). Remote programming of cochlear implants. Otol. Neurotol..

[30] Steps to a Cochlear Implant|American Cochlear Implant Alliance. (n.d.).. https://www.acialliance.org/general/custom.asp?page=StepstoaCochlearImplant.

[31] Nassiri A.M., Marinelli J.P., Sorkin D.L., Carlson M.L. (2021). Barriers to adult cochlear implant care in the United States: An analysis of health care delivery. Semin. Hear..

[32] Drake M., Friedland D.R., Hamad B., Marfowaa G., Adams J.A., Luo J., Flanary V. (2023). Factors associated with delayed referral and hearing rehabilitation for congenital sensorineural hearing loss. Int. J. Pediatr. Otorhinolaryngol..

[33] Nassiri A.M., Saoji A.A., DeJong M.D., Tombers N.M., Driscoll C.L.W., Neff B.A., Haynes D.S., Carlson M.L. (2022). Implementation Strategy for Highly-Coordinated Cochlear Implant Care with Remote Programming: The Complete Cochlear Implant Care Model. Otol. Neurotol..

[34] Albertson E.M., Chuang E., O’MAsta B., Miake-Lye I., Haley L.A., Pourat N. (2021). Systematic Review of Care Coordination Interventions Linking Health and Social Services for High-Utilizing Patient Populations. Popul. Health Manag..

