# Supplementary material for: Advancing Pediatric Cochlear Implant Care Through a Multidisciplinary Telehealth Model: Insights from Implementation and Family Perspectives
Source: Children (Basel). 2025 Dec 26;13(1):39. doi: 10.3390/children13010039 (PMC12839940; doi:10.3390/children13010039)
Supplement: Supplementary file 1 [file children-13-00039-s001.zip › children-4022147-supplementary.pdf]

Supplemental Table S1. Caregiver Technology Use and Comfort

Survey Question	Response Category	Pre-CI (n=11) %(n)	Established CI (n=28) %(n)
**I use a Smartphone on a daily basis.**	Strongly agree / Agree	100% (10)	91.3% (21)
	Disagree / Strongly disagree	0% (0)	8.7% (2)
**I use a computer or tablet on a daily basis.**	Strongly agree / Agree	90.0% (9)	65.2% (15)
	Disagree / Strongly disagree	10.0% (1)	34.8% (8)
**I check my email on a daily basis.**	Strongly agree / Agree	100% (10)	91.3% (21)
	Disagree / Strongly disagree	0% (0)	8.7% (2)
**I send emails on a daily basis.**	Strongly agree / Agree	70.0% (7)	65.2% (15)
	Disagree / Strongly disagree	30.0% (3)	34.8% (8)
**I send text messages on a daily basis.**	Strongly agree / Agree	90.0% (9)	86.9% (20)
	Disagree / Strongly disagree	10.0% (1)	13.1% (3)
**I regularly use Skype or another videoconferencing service.**	Strongly agree / Agree	50.0% (5)	69.6% (16)
	Disagree / Strongly disagree	50.0% (5)	30.4% (7)
**I regularly use Bluetooth technology, or "pair" my devices to one another.**	Strongly agree / Agree	80.0% (8)	78.2% (18)
	Disagree / Strongly disagree	20.0% (2)	21.8% (5)
**People close to me would refer to me as "plugged in."**	Strongly agree / Agree	60.0% (6)	73.9% (17)
	Disagree / Strongly disagree	40.0% (4)	26.1% (6)
**I always have my cell phone at my side.**	Strongly agree / Agree	100% (10)	100% (23)
**Technology is helpful.**	Strongly agree / Agree	100% (10)	100% (23)
**People close to me would refer to me as "tech savvy."**	Strongly agree / Agree	60.0% (6)	73.9% (17)
	Disagree / Strongly disagree	40.0% (4)	26.1% (6)
**If we have technology-related issues in my household, I am the one to address them.**	Strongly agree / Agree	60.0% (6)	69.6% (16)
	Disagree / Strongly disagree	40.0% (4)	30.4% (7)
**If we have technology-related issues in my household, I ask someone else to address them.**	Strongly agree / Agree	40.0% (4)	82.6% (19)
	Disagree / Strongly disagree	60.0% (6)	17.4% (4)
**I am easily frustrated by technology.**	Strongly agree / Agree	30.0% (3)	82.6% (19)
	Disagree / Strongly disagree	70.0% (7)	17.4% (4)
**When something goes wrong with my computer, I can fix it.**	Strongly agree / Agree	70.0% (7)	69.6% (16)
	Disagree / Strongly disagree	30.0% (3)	30.4% (7)
**Technology creates more problems than it solves.**	Strongly agree / Agree	30.0% (3)	91.3% (21)
	Disagree / Strongly disagree	70.0% (7)	8.7% (2)
**When I don't know how to do something on my computer, I can figure it out on my own or with online resources.**	Strongly agree / Agree	100% (10)	95.7% (22)
	Disagree / Strongly disagree	0% (0)	4.3% (1)
**I have patience when it comes to technology-related issues.**	Strongly agree / Agree	100% (10)	95.7% (22)
	Disagree / Strongly disagree	0% (0)	4.3% (1)
**When I want to learn about something on the Internet, I can find it.**	Strongly agree / Agree	100% (10)	100% (23)
**When I encounter a technology-related problem, I give up and walk away.**	Strongly agree / Agree	20.0% (2)	91.3% (21)
	Disagree / Strongly disagree	80.0% (8)	8.7% (2)

Supplemental Table S2a. Time, Work, School, and Care Coordination Burden

Survey Question	Response Category	Pre-CI (n=11) %(n)	Established CI (n=28) %(n)
**How much time did you spend away from home/work when you attended today's appointment?**	0–2 hours	45.5% (5)	92.6% (25)
	3–5 hours	45.5% (5)	3.7% (1)
	6–8 hours	9.1% (1)	3.7% (1)
**Did you have to take time off work to attend today's appointment?**	None	54.5% (6)	85.7% (24)
	≥2 hours	45.5% (5)	14.3% (4)
**What type of time off did you take?**	Unpaid time off	62.5% (5)	75.0% (12)
	Paid time off	37.5% (3)	25.0% (4)
**Did you or your child have to take time off school to attend today's appointment?**	None	72.7% (8)	82.1% (23)
	≥2 hours	27.3% (3)	17.9% (5)
**Did you have to coordinate with someone else to care for other dependents?**	Yes	50.0% (4)	14.3% (4)
	No	50.0% (4)	53.6% (15)
	Not applicable	—	32.1% (9)

Supplemental Table 2b. Caregiver Satisfaction with In-Person Clinic Visit

Survey Question	Response Category	Pre-CI (n=11) %(n)	Established CI (n=28) %(n)
**It was easy for me to attend my in-person clinic visit.**	Strongly agree / Agree	81.8% (9)	51.9% (14)
	Disagree / Strongly disagree	18.2% (2)	48.1% (13)
**It was easy to get to my appointment on time.**	Strongly agree / Agree	63.6% (7)	51.9% (14)
	Disagree / Strongly disagree	36.4% (4)	48.1% (13)
**Overall, how satisfied were you with your in-person clinic visit?**	Very satisfied / Satisfied	100% (11)	77.8% (21)
	Slightly / Very unsatisfied	0% (0)	22.2% (6)
**How do you rate the overall quality of care that you or your child received?**	Excellent / Very good	90.9% (10)	100% (27)
	Fair	9.1% (1)	0% (0)
**My provider spent ________ amount of time with me.**	Just the right amount	90.9% (10)	96.3% (26)
	Too little / Too much	9.1% (1)	3.7% (1)

Supplemental Table S3a. Multidisciplinary Team Satisfaction

Survey Question	Response Category	Pre-CI (n=11) %(n)	Established CI (n=28) %(n)
**How satisfied were you with the overall multi-disciplinary team meeting?**	Extremely satisfied	100% (10)	95% (19)
	Somewhat satisfied	0% (0)	5% (1)
**How important is it for your child's providers to work together?**	Extremely important	100% (10)	100% (20)
**How well do you think the entire team was on the same page about your child's care?**	Extremely	100% (10)	95% (19)
	Somewhat	0% (0)	5% (1)
**Would you prefer to meet with the multi-disciplinary team members individually or as a group?**	Group	90% (9)	50% (10)
	Individually	10% (1)	50% (10)
**Would you want these visits to be remote or in-person?**	Remote	100% (10)	95% (19)
	In-person	0% (0)	5% (1)

Supplemental Table S3b. Caregiver Preferences for Multidisciplinary Team

Survey Question	Response Category	Pre-CI (n=11) %(n)	Established CI (n=28) %(n)
**Which specialists would you like to see again? (Select all that apply)**	Psychology	70% (7)	45% (9)
	Education	70% (7)	80% (16)
	Speech Therapy	90% (9)	65% (13)
	None	30% (3)	55% (11)

Supplemental Table S3c. Caregiver Preferences for Multidisciplinary Team Follow-Up

Survey Question	Response Category	Pre-CI (n=11) %(n)	Established CI (n=28) %(n)
**How often would you like to meet with the multidisciplinary team?**	Every 6 months	80% (8)	55% (11)
	Once a year	10% (1)	20% (4)
	Only when I have concerns	10% (1)	25% (5)
